# Relationship between Degree of Polymeric Ionisation and Hydrolytic Degradation of Eudragit^®^ E Polymers under Extreme Acid Conditions

**DOI:** 10.3390/polym11061010

**Published:** 2019-06-07

**Authors:** Valentina Linares, Cristhian J. Yarce, Juan D. Echeverri, Elkin Galeano, Constain H. Salamanca

**Affiliations:** 1 Departamento de Ciencias Farmacéuticas, Facultad de Ciencias Naturales, Universidad Icesi, Calle 18 No. 122-135, Cali 76003, Colombia; valentina.linares@correo.icesi.edu.co (V.L.); cjyarce@icesi.edu.co (C.J.Y.); 2 Programa de Maestría en Formulación de Productos Químicos y Derivados, Facultad de Ciencias Naturales, Universidad Icesi, Calle 18 No. 122-135, Cali 76003, Colombia; jdecheverri@icesi.edu.co; 3 Grupo de Investigación en Sustancias Bioactivas, Facultad de Ciencias Farmacéuticas y Alimentarias, Universidad de Antioquia, calle 66 No. 53-108, Medellín 050010, Colombia; elkin.galeano@udea.edu.co

**Keywords:** Eudragit^®^ E 100, Eudragit^®^ E PO, cationic polymer, polymeric ionisation degree, polymeric hydrophobic surfaces

## Abstract

The commercial copolymers Eudragit^®^ E 100 and Eudragit^®^ PO are widely used materials in the pharmaceutical field as coating systems. Such materials derived from amino-methacrylate groups under acidulated conditions may acquire an ionisable fraction or undergo hydrolytic degradation of the polymeric structure. This work focused on establishing the chemical, physical, and surface changes of two reprocessed polymeric materials, here named as EuCl-E-100 and EuCl-E-PO, which were obtained from the commercial Eudragit^®^ E 100 and Eudragit^®^ E PO, respectively. The commercial materials were exposed to extreme acid conditions, where the polymers were solubilised and subsequently dried by the refractance window method. The materials obtained were chemically characterised by potentiometric titration, nuclear magnetic resonance spectroscopy (^1^H NMR and ^13^C NMR) in one and two dimensions (COSY, HSQC, and HMBC), infrared spectroscopy, X-ray diffraction, and differential scanning calorimetry. Changes in the physical properties of the materials were evaluated through studies of flowability, compactability, and their ability to gain and lose humidity. Surface thermodynamic studies were carried out through contact angle measurements using the sessile drop method. The results showed that the processed polymeric materials acquired a substantial degree of ionisation without undergoing hydrolysis of the esterified groups. Furthermore, such changes improved the flow characteristics of the material and the solubility in aqueous media at pH > 5, while also maintaining the hydrophobicity degree of the polymeric surface.

## 1. Introduction

Eudragit^®^ polymers are a family of materials derived from poly(methacrylates) widely used in the pharmaceutical field for several purposes [1,2,3,4,5]. Within these materials are Eudragit^®^ E 100 and Eudragit^®^ E PO, which are two commercially available polymers, mainly used like coating materials to prevent the effects of humidity-mediated degradability [5] or to improve the sensory characteristics of pharmaceutical tablets [6]. These materials commonly are referred to as amino methacrylate copolymers (USP/NF) [7] or basic butylated methacrylate copolymers (Ph. Eur.) [8] but are, in fact, copolymers formed from the methacrylate dimethyl aminoethyl, butyl methacrylate, and methyl methacrylate groups in a ratio of 2:1:1 [3]. 

These polymeric materials derived from Eudragit^®^ have been widely used in the pharmaceutical field for several decades as coating systems [6,9,10,11], since these can act as organoleptic correctors [3,12,13], solubility improvers [14,15,16], drug release modifiers [10,11,17,18], and more recently, like the nano-systems stabilisers [16,19,20]. 

However, the main problem of these materials is the low solubility in aqueous media (at pH > 5), which is in fact, a great limitation for the biological and pharmaceutical field, since the most of the physiological pHs are above such a value.

In this regard, the use of Eudragit E^®^ polymers as coating systems has led to employ several physicochemical strategies, which at the same time have their own intrinsic problems. Some of these strategies are the use of (i) organic solvents and (ii) aqueous solutions acidulated. In the first case, the problem lies in the residual contamination, which is generated during the surface coating stage, where the use of organic solvents is necessary to create the polymeric coating solutions [3,13]. 

On the other hand, when acidulated aqueous solutions are employed, the problem lies in the degradability of the polymeric material, where the polymer side chains could be hydrolysed, affecting their capability as a coating system.

**Figure 1:**
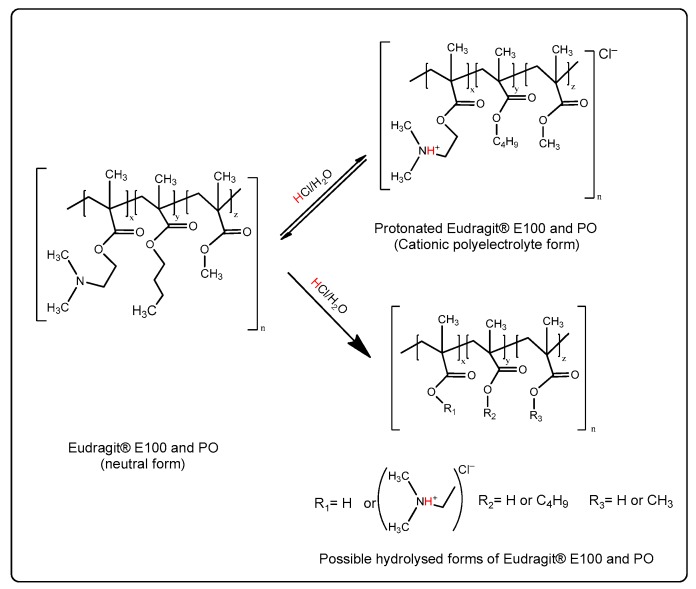
Presumptive scheme of the ionisation and hydrolytic degradation of Eudragit^®^ E-100 and Eudragit^®^ PO under acidic conditions.

In recent years, our laboratory has conducted several research papers on this matter, where it was found that such polymers could be reprocessed to new materials with different physicochemical properties according to the balance between the ionisation degree (protonation) of the dimethyl aminoethyl groups (DMAE) and hydrolysis of the esterified alkyl groups of the polymer backbone, as shown in Figure 1. 

Such ionisation/hydrolysis balance in the polymeric material allows one to improve the solubility in aqueous media at pH > 5, where the material can be used for the development of different nano-particulate systems in aqueous media, such as polymer-drug nanocomplexes [21] or nanoliposomes coated with polymers [22,23].

Nevertheless, when these materials are intended as hydrophobic coating systems on solid surfaces, the loss of the esterified alkyl substituents drives a decrease of the nonpolar component, affecting the traditional application as a coating agent. Therefore, the goal of this research was to establish a reprocessing of the Eudragit E polymeric materials, so that they were soluble in aqueous media at pH > 5, but at the same time, kept their structural integrity in the side alkyl chains.

## 2. Material and Methods

### 2.1. Materials

The commercial polymers Eudragit^®^ E 100 and Eudragit^®^ E PO were acquired from Evonik (Essen, Germany), whereas HCl, NaOH, acetic acid, HClO_4_, isopropanol, and ethylene glycol were purchased from Merck KGaA (Darmstadt, Germany). Type II water was obtained from a purification system (Millipore Elix Essential, Merck KGaA, Darmstadt, Germany). 

### 2.2. Obtention of Polymeric Materials

The processed polymers EuCl-E-100 and EuCl-E-PO where obtained from the commercial forms of Eudragit^®^ E100 and Eudragit^®^ E PO, respectively, under extreme acidity. Briefly, 10 g of Eudragit^®^ E polymer was dissolved in 70 mL of aqueous HCL at 37% *v*/*v*. Subsequently, this mixture was stirred for 1 h at room temperature, during which the system went from a heterodisperse mixture to a homogenous mixture. The homogeneous dispersion was then dried in a refractance window dryer [24] (CEI Robots, Cali, Colombia) at 40 °C for ~4 h. Afterwards, the processed polymeric materials were sieved using a 75 μm mesh (number 200). In the case of Eudragit E^®^ 100, before the dispersion process, the polymer was grounded.

#### Determination of Ionisation Degree and Zeta Potential

The ionisation effect on the DMAE groups as well as the hydrolysis of the esterified groups in the Eudragit E polymers were evaluated by means of potentiometric studies and measurements of the zeta potential in triplicate. First, the amount of DMAE groups present in the commercial and reprocessed polymers of Eudragit^®^ E were determined by means of an acid-base potentiometric titration. For this, 0.2 g of the polymeric material was dissolved in a binary mixture formed from 96 mL of glacial acetic acid and 4 mL of water. Subsequently, this polymer solution was titrated with an aqueous solution of 0.1 N perchloric acid (previously standardised) using a Titrette^®^-Brand automatic titration system (Essex, CT, USA) and a HI931 Automatic Potentiometric Titrator—Hanna Instruments (Limena, Italy). The DMAE groups in the polymers were determinate from the alkali value, which states how many mg of KOH is equivalent to the number of basic groups contained in 1 g of dry substance (DS), as shown in Equations (1) and (2).

(1) $$\%\left(DMAE\right)groups=AV\;\left(\frac{mg\;KOH}{g\;DS}\right)\times 0.1286$$

(2) $$AV=\frac{V_{HClO_{4}}\times 561}{SW\times \%DS}$$

where *V*_*HClO*_4__ is the volume of 0.1 N perchloric acid, *561* correspond to the equivalence between the volume in millilitres of perchloric acid 0.1 N and the mass in milligrams of potassium hydroxide, *SW* is the sample weight and *%DS* is the fraction of dry substrate (dry Eudragit polymer) as a percentage. In this way, the ionisation degree (α) was established from the difference in the DMAE groups percentage before and after polymeric processing under acid conditions, in accordance with

(3) $$\alpha =\frac{\%DMAE\;\left(before\right)\;}{\%DMAE\;\left(after\right)}\times 100$$

Conversely, the hydrolysis of the esterified groups in the Eudragit E polymers was established through the determination of the carboxylic acid percentage % (CA) according to

(4) $$\%\;CA\;groups=\frac{V_{NaOH}\times 430.45}{SW\times \%DS}$$

where *V_NaOH_* is the volume of 0.5 N sodium hydroxide, *430.45* correspond to the amount of methacrylic acid per millilitre of 0.5 N sodium hydroxide, *SW* is the mass of the sample and *%DS* is the percentage of the dry substrate. The potentiometric titration methodology and all these equations were taken from the data sheet for polymer Eudragit E-100™ provided by Evonik Industries. On the other hand, the zeta potential measurements in aqueous media were performed using a Zetasizer Nano ZSP (Malvern Instrument, Worcestershire, UK) at 25 °C and a disposable folded capillary cell (DTS1070). 

### 2.3. Structural Characterisations of Polymeric Materials

#### 2.3.1. FT-IR Characterisation

The structural characterisation of the polymeric materials was performed in an FT-infrared spectrometer (Nicolet 6700, Thermo Fisher Scientific, Waltham, MA, USA). The spectra were recorded by using an attenuated total reflectance Smart iTR™ accessory, where the spectra of the precursor materials, Eudragit^®^ E100 and Eudragit^®^ E PO were compared with the processed materials.

#### 2.3.2. NMR Characterisation

^1^H NMR and ^13^C NMR were recorded on a Bruker Ascend III HD 600 MHz with a 5 mm CryoProbe TCI, using CDCl_3_ and D_2_O as deuterated solvents; TMS and TSP were employed as the chemical shift reference (δ = 0.0 ppm) and internal standard, respectively. Signals were assigned using one- and two-dimensional ^1^H–^1^H COSY, ^1^H–^13^C HMQC and HMBC spectra. 

Non-polar samples solutions were prepared in CDCl_3_ and analysed via ^1^H NMR with a 30° pulse (Bruker zg30 pulse sequence) and polar protonated derivatives were analysed ^1^H NMR with a 90° pulse with water presaturation sequence (Bruker zgpr pulse sequence). All proton spectra were obtained at 298 K, and the delay time was 2 s with eight scans for each spectrum. The spectra were processed using Fourier transform with 64 K data points and a spectral width of 8417.5 Hz.

#### 2.3.3. X-ray Diffraction (XRD)

X-ray diffraction patterns were obtained on an empyrean diffractometer (XPERT Panalytica, Serie II, detector PIXCel3D, 2012, operated at 40 kV) equipped with a monochromatic CuKα (α_1_ = 1.5406 Å; α_2_ = 1.5443 Å).

#### 2.3.4. Thermal Analysis

Thermal studies were carried out on a Q2000 differential scanning calorimeter (DSC; TA Instruments, New Castle, DE, USA) calibrated with indium *T*_m_ = 155.78 °C ∆*H*_m_ = 28.71 J/g. Thus, three modulated heating–cooling cycles from 25 °C (298.15 K) to 300 °C (573.15 K) at a heating rate of 5 °C/min and a modulation of ±0.5 °C every 40s were used. Approximately, 10 mg of each sample was placed on a hermetic crucible with lid, and an empty hermetic crucible was used as a reference.

### 2.4. Physical Characterisation of Polymeric Materials

#### 2.4.1. External Morphology Description

The external morphologies of Eudragit^®^ E 100, Eudragit^®^ E PO, EuCl-E-100 and EuCl-E-PO were observed by micro-display high magnification using a micro-stereoscope (Nikon SMZ1500, Nikon Industries Inc., Melville, NY, USA). The images were processed with the NIS-Elements Advanced Research software (Nikon Industries Inc., Melville, NY, USA). 

#### 2.4.2. Particle Characterisation

Regarding the physical characteristics of polymeric powdered materials, these were studied by means of two parameters corresponding to (i) the angle of repose, and (ii) the Carr and Hausner indexes [25,26]. These parameters show the capability of the powder to flow. Likewise, the flowability degree was obtained through a powder flow tester (Erweka GmbH), whereas the percentage of compressibility was determined using a density meter (Logan Tap-2S) [27]. 

#### 2.4.3. Disintegration and Dissolution Test

The polymeric tablets were made using a homemade tablet press with ¼ inch in diameter flat stainless-steel punches. For each tablet, 500 mg of polymer material were used. Three compression pressures of 200, 300 and 400 psi were applied for 10 s in each tableting. The hardness was determined using a durometer (Logan HDT-400), while the disintegration and qualitative dissolution was determined by an automated disintegrator (Logan USP DST-3) at 37 °C, using 1L of PBA buffer pH: 7.4.

#### 2.4.4. Humidity Loss and Gain Studies

The humidity gain was determinate from 2 g of polymer that had previously been dried at 40 °C for 24 h. Each polymeric material was then taken to a stability chamber (40 ± 5°C and relative humidity of 75% ± 2%), where weight measurements were made until a constant value was obtained. For humidity loss, 2 g of polymer was also used, but in this case, a humidity balance (Ohaus MB35) was used with a temperature range between 100 and 160 °C.

### 2.5. Thermodynamics Surface Characterisation

#### 2.5.1. Contact Angle Measurements

The commercial polymer Eudragit^®^ E 100, which was purchased as pellets, was ground to form granules of smaller size that are easier to compact. The rest of the polymers were already easily compactable powders. To generate the polymer compact surfaces, 200 mg of material was used, and a compression force of 400 psi was applied for 10 s. Once the compact surfaces of the polymeric materials were formed, the static contact angle was determined immediately. For each system, the sessile drop method [28,29] was carried out using a contact angle metre (OCA15EC Dataphysics Instruments, Filderstadt, Germany) with software controller (SCA20 and SCA21 version 4.5.14). The data capture was recorded using an IDS video camera, where the information obtained was taken over 400–800 frames as a reference point. Moreover, the contact angle capture point was defined when the reflection from the incident drops disappeared completely (approximately 1seg, since its exit from the dispensing system). A fixed height of 1 cm was used for dropping. The volume added was in the range 0.005–0.015 mL. Each measurement was performed at 22 ± 1 °C temperature and at a relative humidity of 60% ± 5%, as determined with a digital Thermo hygrometer (HTC-1, Bestone measuring instrument, Guangdong, China). The contact angle was measured in triplicate over the surface of the tablets using type II water. 

#### 2.5.2. Surface Free Energy (SFE) Determination

The SFE were determined by using the OWRK model [30,31,32,33]. For this, isopropanol, ethylene glycol and water as the liquid tests were used. This model describes the solid SFE of the polymeric materials in terms of the polar (hydrophilic) (SFE_p_) and dispersive (hydrophobic) (SFE_d_) contributions.

## 3. Results and Discussion

### 3.1. Obtention of Polymeric Materials

#### Determination of Ionisation Degree and Zeta Potential

The results of the percentage of DMAE groups, the ionisation degree and the zeta potential values for polymers Eudragit^®^ E100 and Eudragit^®^ E PO, as well as their processed forms EuCl-E-100 and EuCl-E-PO are summarised in Table 1.

It was found that the percentage of DMAE groups in the commercial polymers Eudragit^®^ E100 and Eudragit^®^ E PO were 20.9% ± 0.1% and 19.5% ± 0.2%, respectively, which are very close to the values described in the technical sheet of both polymers (20.8–25.5%) [3,34]. Furthermore, it was found that for these polymers, the zeta potential values could not be determined as they formed large non-dispersible aggregates in aqueous medium.

On the other hand, the processed polymers EuCl-E-100 and EuCl-E-PO showed different percentages of dimethylamine groups of 7.9% and 7.5%, respectively, which are equivalent to an ionisation degree of ~38% in both materials. These results suggest that during extreme acidification, the DMAE groups do not completely ionise, forming a thermodynamic equilibrium between the basic form of the dimethylamine substituent and the protonated form, corresponding to the DMAE chloride. This effect was also inferred by the difference in the zeta potential values of the commercial polymers (neutral form) and their processed forms (ionised form), going from undetermined values to ~ +48 mV, suggesting an increase in the polymeric surface charge. 

On the contrary, it is noteworthy that during this process, there was no evidence for the presence of carboxylic acid groups, suggesting that hydrolysis of the alkyl esterified groups did not occur. This is a rather surprising result because at such acid conditions, it is expected that the alkyl esterified groups would undergo some degree of hydrolysis. Besides, previously we have shown that this class of polymers underwent both ionisation of the DMAE group and hydrolysis of the esterified groups [21]. The difference between these two contrasting results is attributed to a dialysis step, which was not performed in the current study, but meant that the polymeric material was kept in an aqueous solution for 14 days, long enough to promote hydrolysis. Therefore, the extended time seems to significantly affect the ionisation/hydrolysis balance in polymers of Eudragit^®^ E and so should be studied further in the future.

### 3.2. Structural Characterisation of Polymer Materials

#### 3.2.1. FT-IR Characterisation

**Figure 2:**
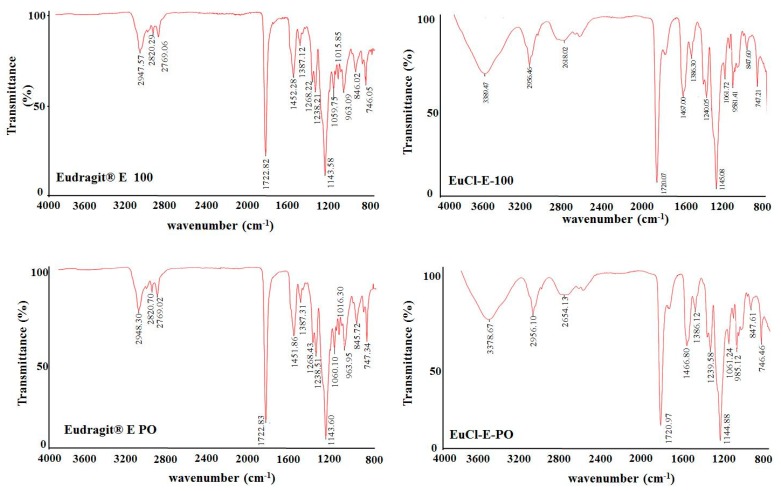
FTIR spectra of polymers derived from Eudragit E-100.

Figure 2 shows the IR spectra for the commercial polymers Eudragit^®^ E100 and Eudragit^®^ E PO and the processed materials EuCl-E-100 and EuCl-E-PO. 

In the FTIR spectra of all the polymeric materials studied, characteristic bands of the esterified groups at 1150–1190, 1240 and 1270 cm^−1^ were observed, as well as the vibration of the carbonyl group of the ester group at 1730 cm^−1^. In addition, vibrations of the hydrocarbon chain were observed at 1385, 1450–1490 and 2950 cm^−1^. However, the most important signals are observed between 2770 and 2820 cm^−1^, which correspond to the DMAE groups present in all the polymers, and a broad signal exclusive of the processed materials at approximately 3435 cm^−1^ due to R−N^+^–H stretches of the protonated DMAE group (ammonium salt). These results are very interesting because they show that the ionisation process of the DMAE groups is incomplete and that in the processed polymers, the DMAE groups are in a thermodynamic equilibrium between their neutral and ionised forms. Likewise, it was found that the IR spectra of the processed materials did not reveal the wide and intense signal typical of O−H stretches, suggesting that during the treatment with extreme acid, the polymeric materials of Eudragit^®^ E did not suffer the hydrolytic effect of the esterified groups, which agrees with the observed results from the potentiometric titrations.

#### 3.2.2. NMR Characterisation

**Figure 3:**
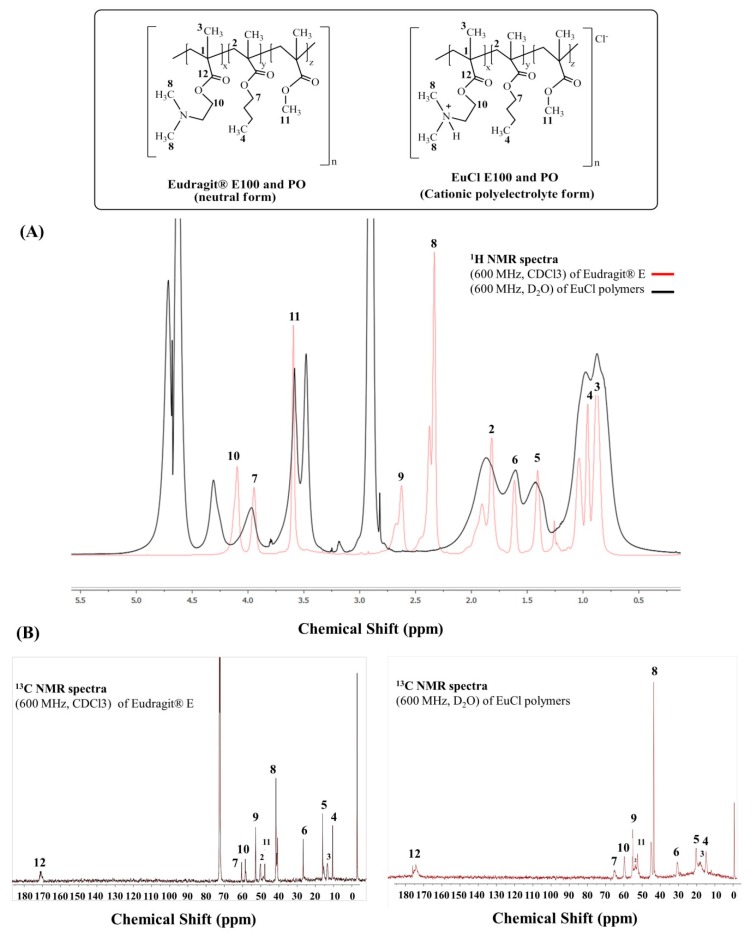
Comparison of the (**A**) proton and (**B**) carbon NMR spectra of Eudragit^®^ and its processed forms.

The ^1^H and ^13^C NMR spectral data confirmed the polymers Eudragit^®^ E 100 and Eudragit^®^ E PO, and their processed forms EuCl-E-100 and EuCl-E-PO had identical chemical structures for each respective family of material. In the proton spectrum, the methyl ester branches are a singlet between 3.51 and 3.67 ppm, the dimethyl amine groups are a singlet with a shoulder between 2.26 and 2.56 ppm, and the methyl groups of the butyl branches are upfield between 0.90 and 1.10 ppm. All proton signals were assigned and corroborated by HSQC and HMBC analyses (Supplementary file). Table 2 summarises the ^1^H and ^13^C peak assignments for the polymers. The protonated Eudragit^®^ in D_2_O undergoes a solvent effect that generates broad peaks and an upfield shift. Protonation of the amine group in the polar derivatives of Eudragit^®^ induces a smaller downfield shift of H-9 due to the electric field shift theory and anisotropic effect (Figure 3). All NMR spectra are available in the supplementary material.

#### 3.2.3. XRD and DSC Analyses

**Figure 4:**
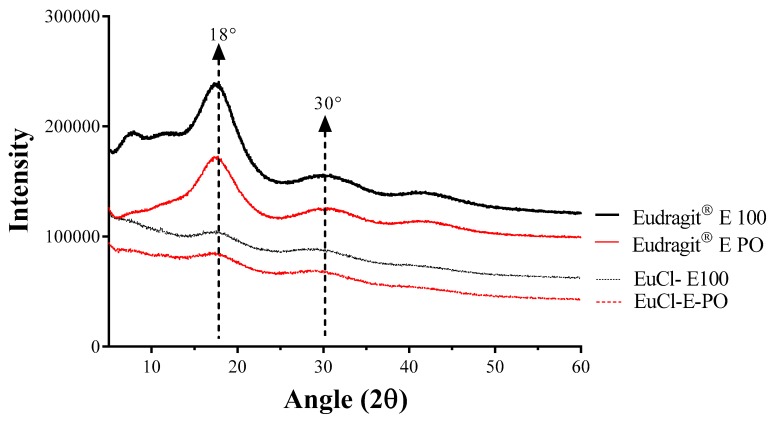
X-ray powder diffractograms of polymers derived from Eudragit^®^ E.

Figure 4 shows the X-ray powder diffractograms for the commercial polymers Eudragit^®^ E100 and Eudragit^®^ E PO and the processed materials EuCl-E-100 and EuCl-E-PO. In the case of the processed polymers, less intense peak patterns are observed, which is typical of amorphous and polymeric materials [35,36,37]. Also, the results show that the acidification process significantly affects the polymeric amorphousness in the materials regardless of the difference in the signals between the precursors and the obtained polymers. Moreover, the XRD behaviour was very similar in both polymeric commercial presentations, and there was no marked difference in the powder and pellet forms.

**Figure 5:**
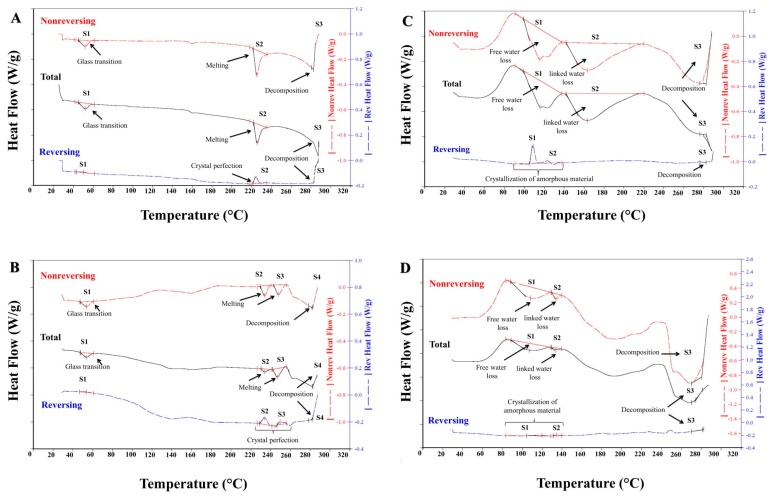
Modulated differential scanning calorimeter (DSC) of (**A**) Eudragit^®^ E 100; (**B**) Eudragit^®^ E PO, (**C**) EuCl-E-100 and (**D**) EuCl-E-PO.

On the other hand, Figure 5 shows the modulated DSC thermograms, whilst Table 3 summarizes the thermal events generated during the first heating cycle applied to each polymeric material. Such thermograms are presented in relation to three types of heat, defined as total heat flow (continuous black line), non-reversible heat flow (red dotted line) and reversible heat flow (blue dotted line) [38,39]. In contrast, the thermograms of the third heating cycle are shown in the supplementary file. 

According to the total energy flow, the thermograms of the Eudragit^®^ E 100 and PO polymers (Figure 5A,B) described three signals corresponding to (i) a glass transition (ii) a fusion process and (iii) a material decomposition. Regarding the glass transition, it was found that it is very similar in both polymers at approximately 50 °C, while the signals of the fusion process exhibited different behaviours depending on the polymer material. In the case of Eudragit^®^ E 100, it was found a signal at approximately 225 °C, whilst for the Eudragit^®^ E PO polymer, two signals were observed at 230 and 242 °C. Likewise, the signals of the fusion process given in the reversible heat flow, exposed a crystallization reorganization event previous to the fusion zone at 224 °C for the Eudragit^®^ E 100 and at 234 and 250 °C for the Eudragit^®^ E PO. On the other hand, the third signal corresponding to the degradation of the material was observed around 282 °C, which was corroborated by means of a change of colour in the polymer material, going from a white to a brown colour as shown in the supplementary file.

In relation to the processed materials EuCl-E-PO and EuCl-E-100 (Figure 5C,D), a very similar thermal behaviour was appreciated among them, but different from that observed with the precursor materials. In this case, the thermograms described three signals corresponding to (i) weakly bound water loss between 107–116 °C, (ii) a linked water loss between 134–163 °C and (iii) a fusion-decomposition around of 282 °C. In addition, the DSC modulated experiment showed by means of the reversible heat cycle that prior to the loss of water in the processed materials, there is a structural rearrangement in the polymers (crystallization of the amorphous material). 

Therefore, the results of DRX and DSC show that during the processing of the materials a change in the polymer features is generated, becoming more amorphous materials.

### 3.3. Characterisation of Powder Polymeric Materials

#### 3.3.1. Powder Morphology and Shape

**Figure 6:**
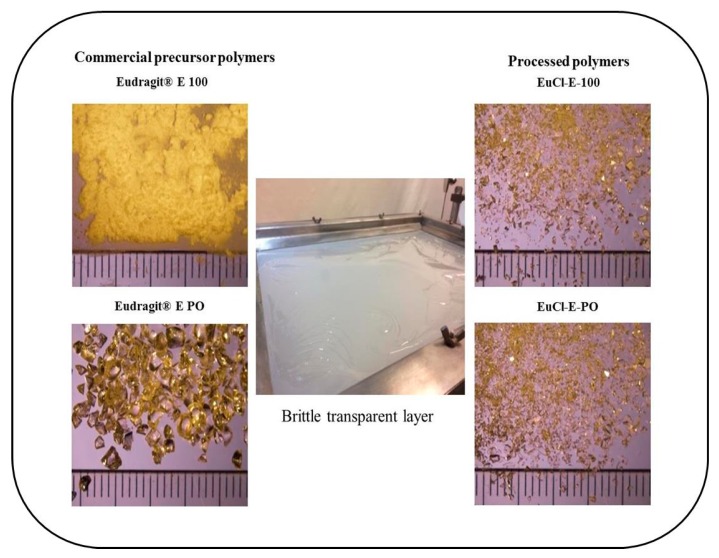
Micrograph images of polymers derived from Eudragit^®^ E.

The polymeric materials Eudragit^®^ E 100 and Eudragit^®^ E PO showed characteristics as described in the technical sheets of both polymers (Figure 6). In the case of Eudragit^®^ E 100, colourless to yellow-tinged granules were observed, whereas Eudragit^®^ E PO was a very fine white powder. Both commercial polymers exhibited a typical amino-like odour. On the other hand, the processed polymeric materials EuCl-E-100 and EuCl-E-PO formed a brittle transparent layer, which was ground and sieved to yield uniform particulate materials. 

#### 3.3.2. Flowability Assays

The particle characterisation results are summarised in Table 4. 

According to the guidelines established in the USP 41/NF35 [7], the Carr and Hausner indexes for the Eudragit^®^ E 100 and Eudragit^®^ E PO commercial polymers presented values between 24% and 6% and 1.3% and 1.1%, respectively, suggesting that the flowability is ranged from good to poor. However, the angle of repose ranged from 7.4° to 30°, suggesting excellent and slight flowability properties, respectively. On the contrary, what processed polymers showed general trend with appropriate particle flow properties.

#### 3.3.3. Humidity Loss and Gain Study

**Figure 7:**
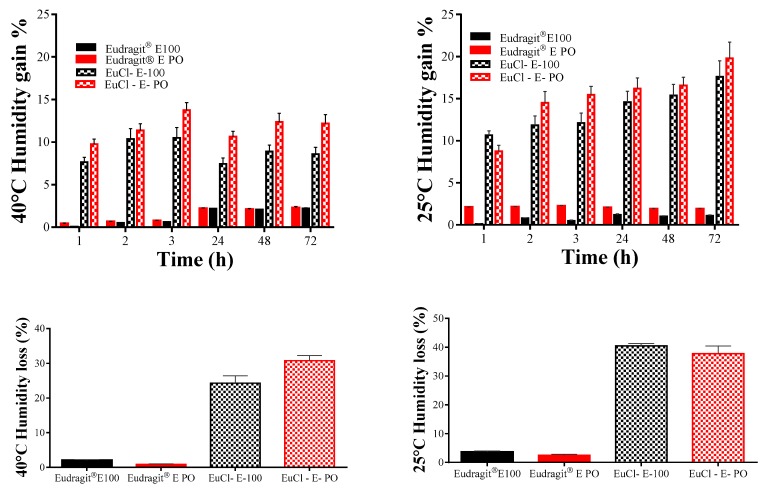
Loss and gain of humidity by polymers derived from Eudragit^®^ E.

The capability of Eudragit^®^ E 100 and Eudragit^®^ E PO, and their processed forms EuCl-E-100 and EuCl-E-PO to lose or gain humidity is presented in Figure 7. 

The results show that the Eudragit^®^ E polymers do not gain or lose humidity significantly, which is consistent with the prevalent hydrophobic nature of such polymers. Conversely, the processed polymeric materials showed a marked tendency to gain or lose moisture, which is attributed to the fact that moisture is adsorbed on the surfaces of the polymeric salts of EuCl-E-100 and EuCl-E-PO. Therefore, these results indicate that these kinds of polymeric salts trap and bind water easily generating hydrated polymeric forms, which is consistent with the results previously obtained by DSC.

#### 3.3.4. Disintegration and Dissolution Test

Table 5 summarizes the results of hardness, disintegration time and qualitative description of the dissolution process of the polymeric tablets. The results of hardness and disintegration time for each polymeric tablet, showed that the increase in the compression force leads to an increase in both parameters. However, the values of disintegration time were very long for commercial polymers and very short for processed polymers. In addition, it was observed that the commercial polymeric materials did not dissolve in the aqueous medium at pH: 7.4, while the processed polymeric materials did dissolve completely. This result is very interesting, considering that the polymers of Eudragit E^®^ have shown a limited solubility in aqueous media above Ph > 5. Therefore, the modification of the materials allows for improvement of such physicochemical properties.

### 3.4. Surface Polymer Characterisation

**Figure 8:**
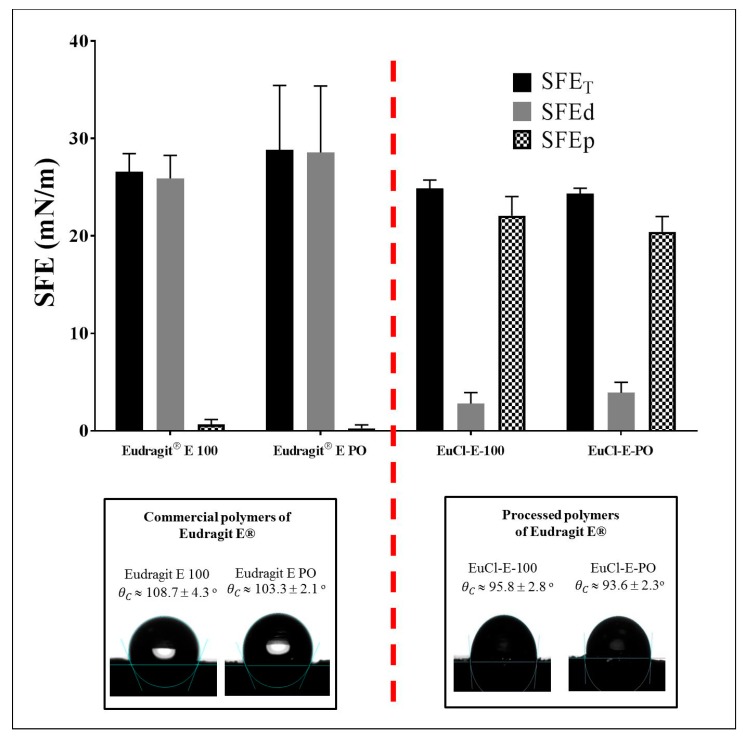
Variation in surface free energy and contact angle between ultra-pure water and the polymers derived from Eudragit E-100.

The results of the contact angle (θ*c*) between each polymeric material and ultra-pure water was studied and the results are shown in Figure 8. These results are very interesting as they show that the hydrophobicity of the polymeric surfaces slightly decreases after the acidification process in aqueous medium. Likewise, it was observed that the contact angle values for Eudragit^®^ E 100 (θc~108°) are slightly larger than Eudragit^®^ E PO (θc~103°), suggesting that the polymeric surface is a little more hydrophobic. These results are coherent if they are related to observations from the humidity gain studies, where such polymers showed a very low ability to trap moisture. 

On the other hand, the processed polymers EuCl-E-100 and EuCl-E-PO showed values of θc around 96° and 94° respectively. This is a very interesting result considering that at values of θc > 90° [28,40,41], the wettability phenomenon on the surface does not happen and so, the polymer modification process did not affect its capability to generate hydrophobic surfaces. This phenomenon is best described according to the SFE, where it is possible to appreciate that the dispersive component of SFE is higher in the Eudragit^®^ E polymers than in the processed polymeric salts, where a decrease in the dispersive component and an increase in the polar component is observed. 

All of these results are coherent if they are related to the previous results; (i) formation of polymer-solvent interactions due to ionisation of the DMAE groups, (ii) change in polymer amorphicity, (iii) capability to imbibe water in the form of hydrated polymers and (iv) ability to generate surfaces with different degrees of hydrophobicity. In this way, the results show that during the exposure to extreme acid conditions, these materials acquire new characteristics that could be used to modulate the solubility in aqueous media (even to pH > 5), maintaining the ability to form hydrophobic surface films.

## 4. Conclusions

The results of the potentiometric study, as well as IR and NMR, corroborated the formation of ionic groups corresponding to a quaternary ammonium salt in the DMAE group. Besides, it was found that during the process of acidification in aqueous medium, the polymeric material does not completely ionise, and the alkyl-esterified groups are not hydrolysed. On the other hand, the XRD results showed that the modification process of the Eudragit E^®^ polymers affected the amorphous characteristics of the precursor material; whereas the results of the thermal studies showed that such materials undergo a structural rearrangement process. Also, it was found that these processed polymers showed a high capability to bind water, which increases as the polymer ionisation degree rises. Each of the polymeric materials showed flowability from good to excellent due to the morphology of regular spheres obtained by the drying process. On the other hand, the contact angle and SFE measurements showed that the modification process slightly affected the polymeric surfaces where the same characteristics of surface hydrophobicity in the co-processed material were practically maintained.

## Figures and Tables

**Table 1:** Ionisation degree obtained for the polymeric materials derived from Eudragit E-100.

Polymeric System	DMAE Groups ± SD (%)	Carboxylic Acid Percentage (%)	Ionisation Degree (%)	Zeta Potential ± SD (mV)
Eudragit^®^ E 100	20.9 ± 0.1	0	0	undetermined
Eudragit^®^ E PO	19.5 ± 0.2	0	0	undetermined
EuCl-E-100	7.9 ± 0.1	0	38	+47.7 ± 1.0
EuCl-E-PO	7.5 ± 0.3	0	38	+47.9 ± 0.3

* The potentiometric titration profile and the zeta potential results for the Eudragit^®^ E polymeric materials are shown in the Supplementary Materials.

**Table 2:** Summary data for the proton and the carbon NMR spectra of Eudragit^®^ and its processed forms.

Position	Eudragit^®^ E100 and PO δ (ppm) CDCl_3_		Form EuCl-E-100 and PO δ (ppm) D_2_O	
	^1^H	^13^C	^1^H	^13^C
1	-	-	-	74.2
2	1.81 br s	54.2	1.86 br s	56.1
3	0.87 br s	16.7	0.80 br s	19.4
4	0.96 br s	13.8	0.87 br s	16.0
5	1.40 br s	19.3	1.35 br s	21.6
6	1.61 br s	30.2	1.59 br s	32.2
7	3.94 br s	64.8	3.97 br s	67.9
8	2.34 (br s. N-CH_3_)_2_	45.1	2.89 br s	45.7
9	2.63 br s	57.0	4.30 br s	62.3
10	4.10 br s	62.7	3.47 br s	57.7
11	3.59 s	51.8	3.58 br s	54.9
12	-	177.9	-	180.4

**Table 3:** Summary data for the modulated DSC analysis for Eudragit^®^ polymers and EuCl derivatives.

Polymer Material	Heat Flow (W/g)	Signal	Signal Type	Onset (°C)	Peak (°C)	Enthalpy (J/g)
Eudragit^®^ E 100	Total	S1	glass transition	44.93	51.89	4.28
		S2	melting point	222.11	225.64	10.32
		S3	decomposition	287.84	-	-
	Reversible	S1	glass transition	42.90	50.53	0.35
		S2	crystal perfection	221.24	224.60	2.89
		S3	decomposition	284.33	-	-
	Non-reversible	S1	glass transition	44.78	51.76	4.63
		S2	melting point	221.62	225.26	13.20
		S3	decomposition	282.73	-	-
Eudragit^®^ E PO	Total	S1	glass transition	47.59	53.60	2.63
		S2	melting amorphous	230.24	234.10	1.36
		S3	melting crystal	242.14	246.98	4.73
	Reversible	S4	decomposition	282.66	-	-
		S1	glass transition	46.72	54.02	0.34
		S2	crystallization	229.01	233.97	3.54
	Non-reversible	S3	crystal perfection	246.51	249.55	0.94
		S4	decomposition	271.73	-	-
		S1	glass transition	47.51	53.64	2.95
EuCl-E-100	Total	S1	free water loss	99.24	115.67	55.92
		S2	linked water loss	141.79	163.15	98.05
		S3	decomposition	282.42	-	-
	Reversible	S1	crystal perfection	105.31	108.74	7.70
		S2	crystallization	124.27	129.13	1.85
		S3	decomposition	282.40	-	-
	Non-reversible	S1	free water loss	99.19	114.83	63.59
		S2	linked water loss	141.79	163.35	102.30
		S3	decomposition	282.71	-	-
EuCl-E-PO	Total	S1	free water loss	88.67	107.38	42.70
		S2	linked water loss	129.40	133.76	3.80
		S3	decomposition	274.42	-	-
		S1	crystal perfection	104.42	119.76	2.01
	Reversible	S2	crystallization	131.19	134.52	0.62
		S3	decomposition	281.21	-	-
		S1	free water loss	88.35	108.39	44.71
		S2	linked water loss	129.70	133.76	4.42
	Non-reversible	S3	decomposition	280.54	-	-
		S1	free water loss	88.67	107.38	42.70
		S2	linked water loss	129.40	133.76	3.80
		S3	decomposition	274.42	-	-

**Table 4:** Flowability data for polymeric material derived from Eudragit E^®.^

Polymer Material	Repose Angle (°) ± SD	Carr Index (%) ± SD	Hausner Index (%) ± SD
Eudragit^®^ E 100	28 ± 0.5	24.0 ± 0.8	1.3 ± 0.05
Eudragit^®^ E PO	7.4 ± 1.3	5.9 ± 0.2	1.1 ± 0.03
EuCl-E-100	15.2 ± 0.6	10.9 ± 0.2	1.1 ± 0.04
EuCl-E-PO	13.3 ± 1.1	10.8 ± 0.3	1.1 ± 0.03

**Table 5:** Results of hardness, disintegration time and formation of homogenous phase of polymeric tablets elaborated with Eudragit^®^ E and EuCl-E materials.

Polymer Material	Applied Pressure (psi)	Tablet Hardness (kp)	Disintegration Time (h:min)	Formation of a Homogeneous Phase
Eudragit^®^ E 100	200	>20	>8h	No
	300			
	400			
Eudragit^®^ E PO	200	2.17 ± 0.13	2:26	No
	300	2.57 ± 0.25	4:75	
	400	3.69 ± 0.43	6:50	
EuCl-E-100	200	10.50 ± 0.20	00:08:	Yes
	300	10.58 ± 1.11	00:10	
	400	11.06 ± 0.17	00:11	
EuCl-E-PO	200	5.40 ± 0.47	00:07	Yes
	300	5.52 ± 0.37	00:10	
	400	5.59 ± 0.47	00:11	

## Supplementary Materials

The supplementary materials are available online at https://www.mdpi.com/2073-4360/11/6/1010/s1.
