# Supplementary material for: Relationship between Degree of Polymeric Ionisation and Hydrolytic Degradation of Eudragit® E Polymers under Extreme Acid Conditions
Source: Polymers (Basel). 2019 Jun 7;11(6):1010. doi: 10.3390/polym11061010 (PMC6630213; doi:10.3390/polym11061010)
Supplement: Supplementary file 1 [file polymers-11-01010-s001.pdf]

Supplementary Material

Herein, we presented a series of graphics that are not included in the paper. This material supports the experimental work of our investigation.

1. *Determination of ionisation degree*

The results of the percentage of DMAE groups, the ionisation degree and the zeta potential values for polymers Eudragit® E 100 and Eudragit® E PO, as well as their processed forms EuCl-E-100 and EuCl-E-PO are presented as potentiometric titration profiles.

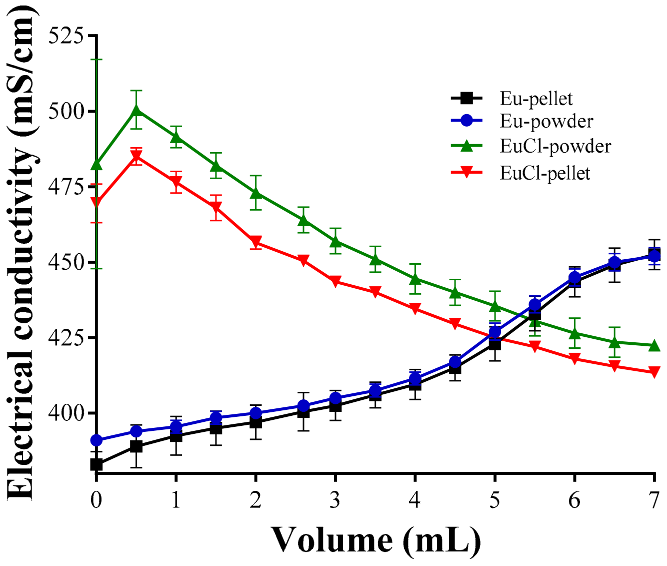

Figure S1. Potentiometric curves obtained for the polymeric materials derived from Eudragit E-100.

The formation of the ionised species of the EuCl polymers were observed by a qualitative variation in the solubility of the Eudragit® E polymers, which changed from a heterogeneous mixture into a homogeneous dispersion, due to the protonation of the DMAE groups. In the potentiometric titrations study of the Eudragit E 100 polymers a single inflection point corresponding to the presence of the DMAE groups in the polymeric backbone was observed.

2. Determination of zeta potential.

For the Eudragit® salt derivatives.

Zeta Potential Report
v2.3

Malvern

Malvern Instruments Ltd - © Copyright 2008

Sample Details

**Sample Name:**	EuCl E 100
**SOP Name:**	Z 040817.sop
**General Notes:**	

**File Name:**	EuCl E 100 .dts	**Dispersant Name:**	Water
**Record Number:**	71	**Dispersant RI:**	1,330
**Date and Time:**	martes, 05 de septiembre de 2...	**Viscosity (cP):**	0,8872
		**Dispersant Dielectric Constant:**	78,5

System

**Temperature (°C):**	25,0	**Zeta Runs:**	30
**Count Rate (kcps):**	2003,8	**Measurement Position (mm):**	2,00
**Cell Description:**	Clear disposable zeta c...	**Attenuator:**	10

Results

			Mean (mV)	Area (%)	St Dev (mV)
**Zeta Potential (mV):**	47,7	**Peak 1:**	44,4	82,7	5,19
**Zeta Deviation (mV):**	10,7	**Peak 2:**	68,3	14,5	6,01
**Conductivity (mS/cm):**	0,757	**Peak 3:**	23,7	1,9	3,24
**Result quality**	Good				

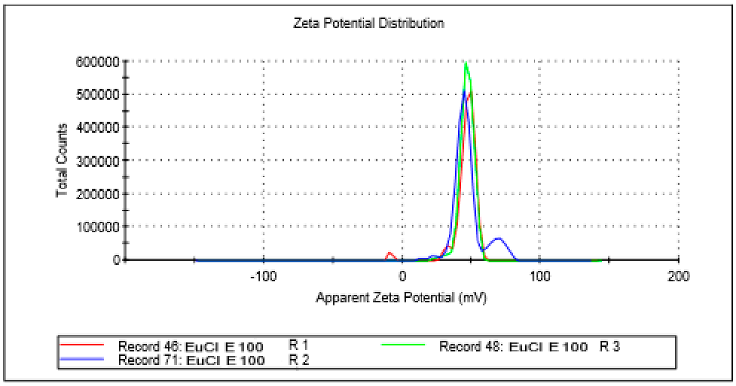

Malvern Instruments Ltd
www.malvern.com

Zetasizer Ver. 7.11
Serial Number : MAL1120280

Record Number: 71
07 mar. 2019 3:02:33 p. m

Figure S2. Zeta potential curves for EuCl– E-100.

Zeta Potential Report
v2.3

Malvern

Malvern Instruments Ltd - © Copyright 2008

Sample Details

Sample Name: EuCl E PO

SOP Name: Z 040817.sop

General Notes:

**File Name:**	EuCl E PO.dts	**Dispersant Name:**	Water
**Record Number:**	101	**Dispersant RI:**	1,330
**Date and Time:**	martes, 05 de septiembre de 2...	**Viscosity (cP):**	0,8872
		**Dispersant Dielectric Constant:**	78,5

System

**Temperature (°C):**	25,0	**Zeta Runs:**	30
**Count Rate (kcps):**	189,0	**Measurement Position (mm):**	2,00
**Cell Description:**	Clear disposable zeta c...	**Attenuator:**	8

Results

			Mean (mV)	Area (%)	St Dev (mV)
**Zeta Potential (mV):**	47,9	**Peak 1:**	49,1	98,8	6,38
**Zeta Deviation (mV):**	7,43	**Peak 2:**	88,0	0,8	1,55
**Conductivity (mS/cm):**	1,47	**Peak 3:**	74,7	0,3	1,84
**Result quality**	Good				

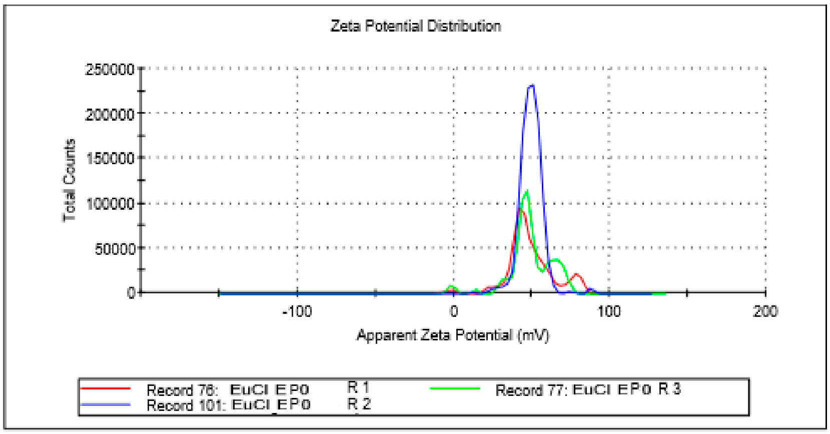

Figure S3. Zeta potential curves for EuCl-E-PO.

3. *Structural characterisation of polymer materials: NMR spectra for the Eudragit® E derived materials.*

^{1}H and ^{13}C NMR spectra are presented; the experiments that were carried out were ^{1}H–^{1}H COSY, ^{1}H–^{13}C HMQC and HMBC.

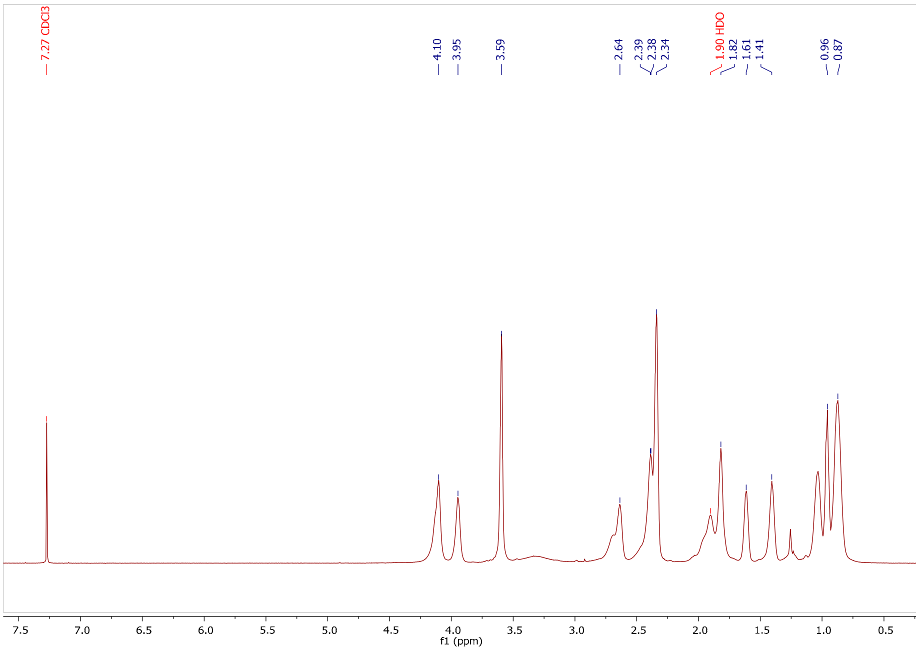

Figure S4. ^{1}H NMR spectra (600 MHz, $CDCl_3$) of Eudragit® E 100.

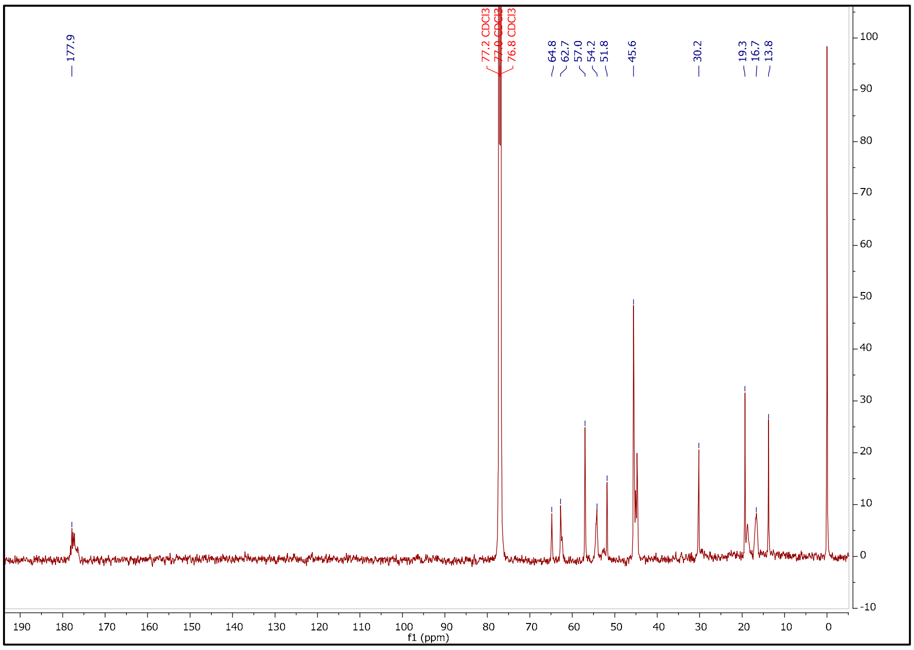

Figure S5. ^{13}C NMR spectra (600 MHz, $CDCl_3$) of Eudragit® E 100.

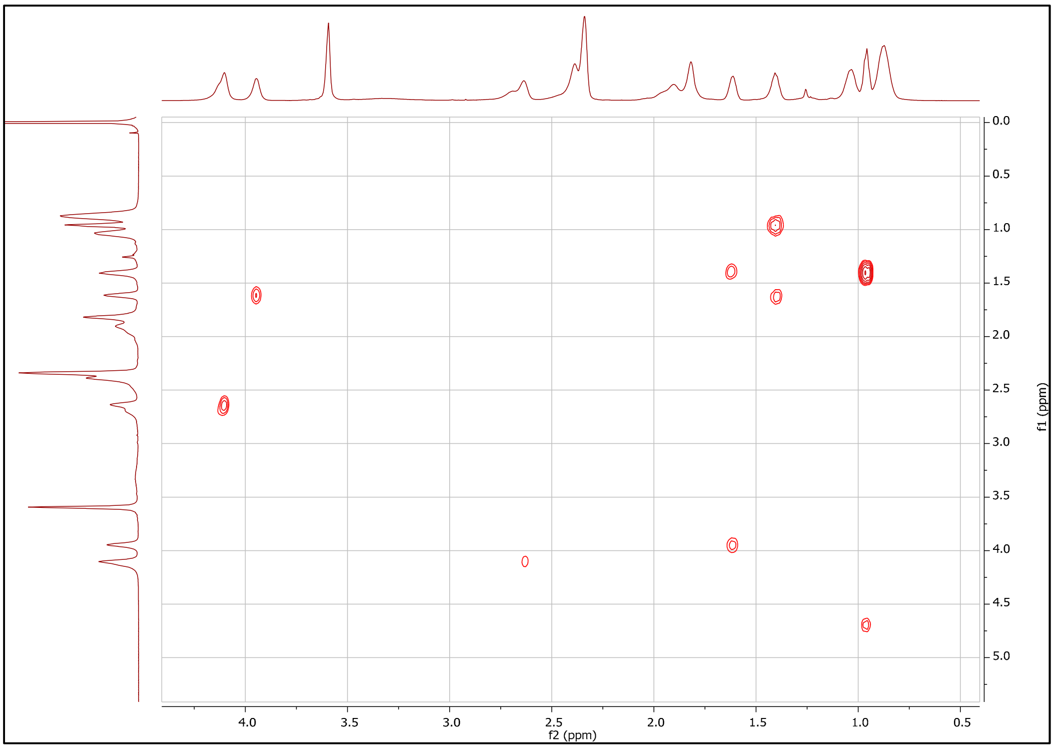

Figure S6. COSY NMR spectra (600 MHz, $CDCl_3$) of Eudragit® E 100.

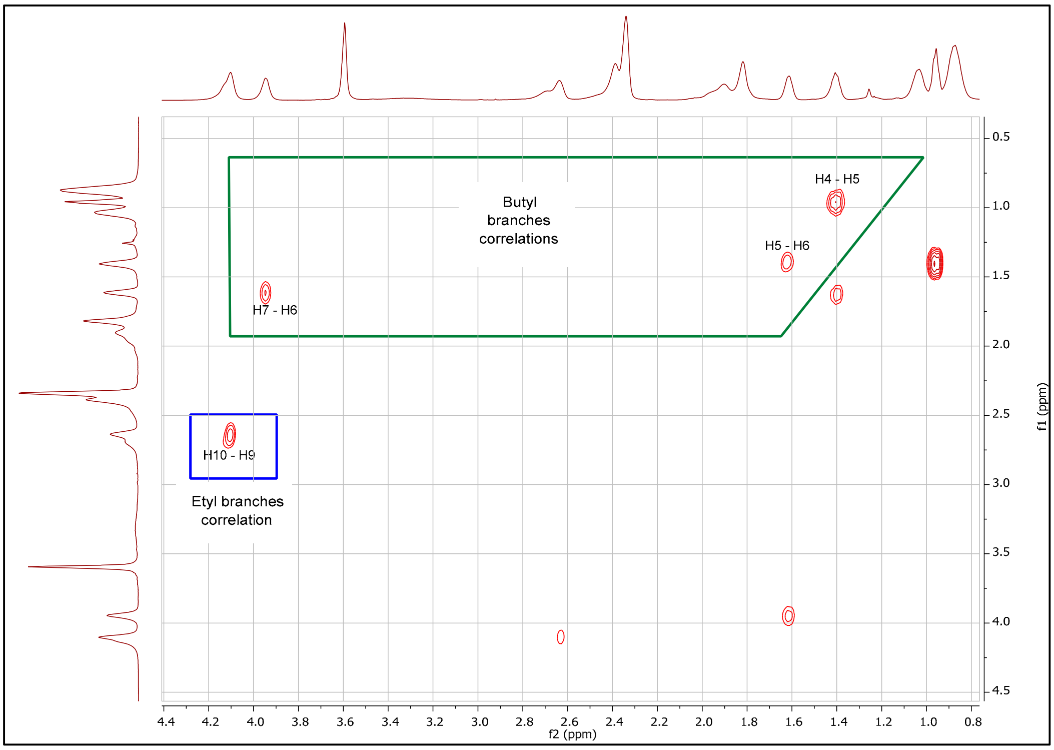

Figure S7. ZOOM COSY NMR spectra (600 MHz, $CDCl_3$) of Eudragit® E 100.

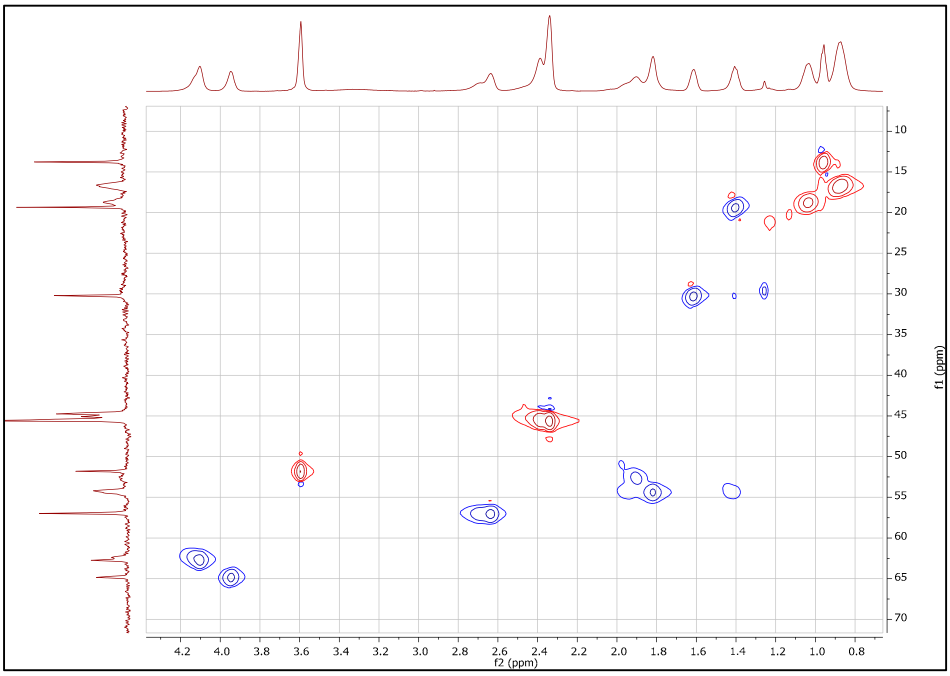

Figure S8. HSQC NMR spectra (600 MHz, $CDCl_3$) of Eudragit® E 100.

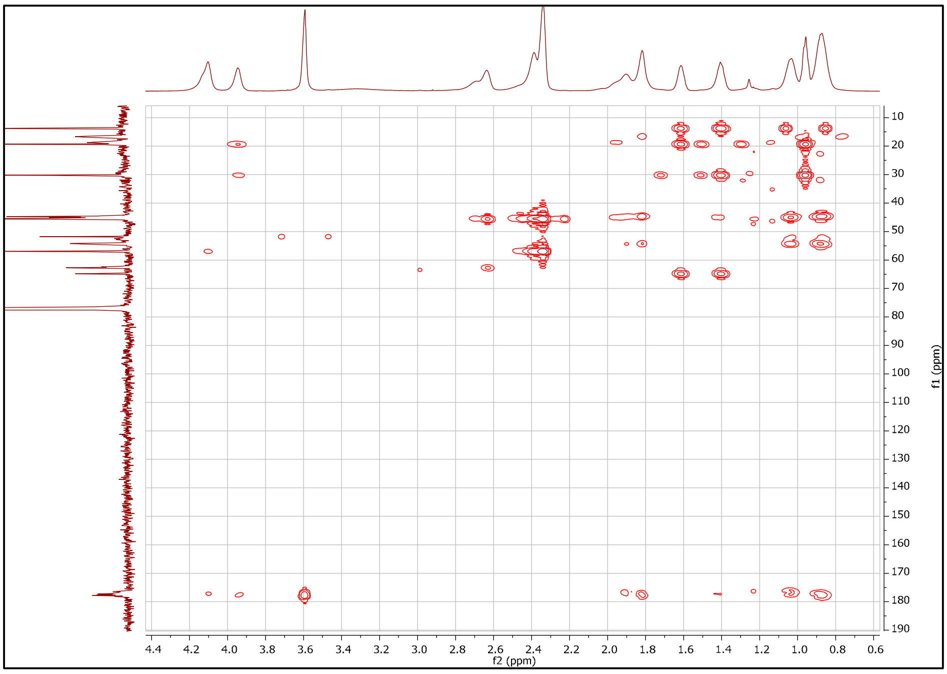

Figure S9. HMBC NMR spectra (600 MHz, $CDCl_3$) of Eudragit® E 100.

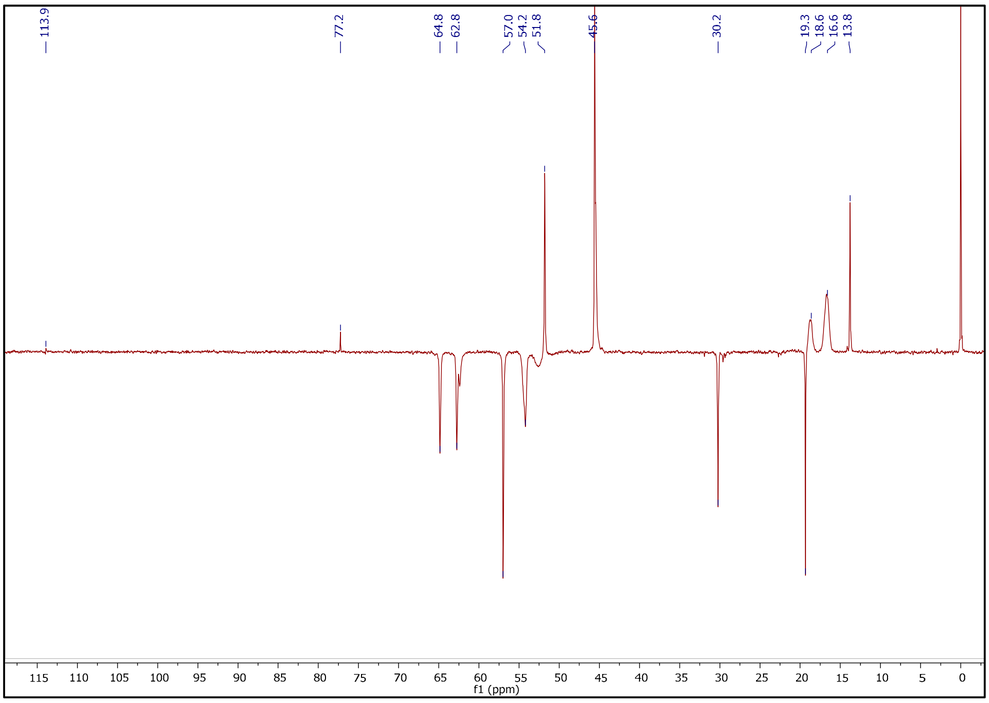

Figure S10. DEPT-135 NMR spectra (600 MHz, $CDCl_3$) of Eudragit® E 100.

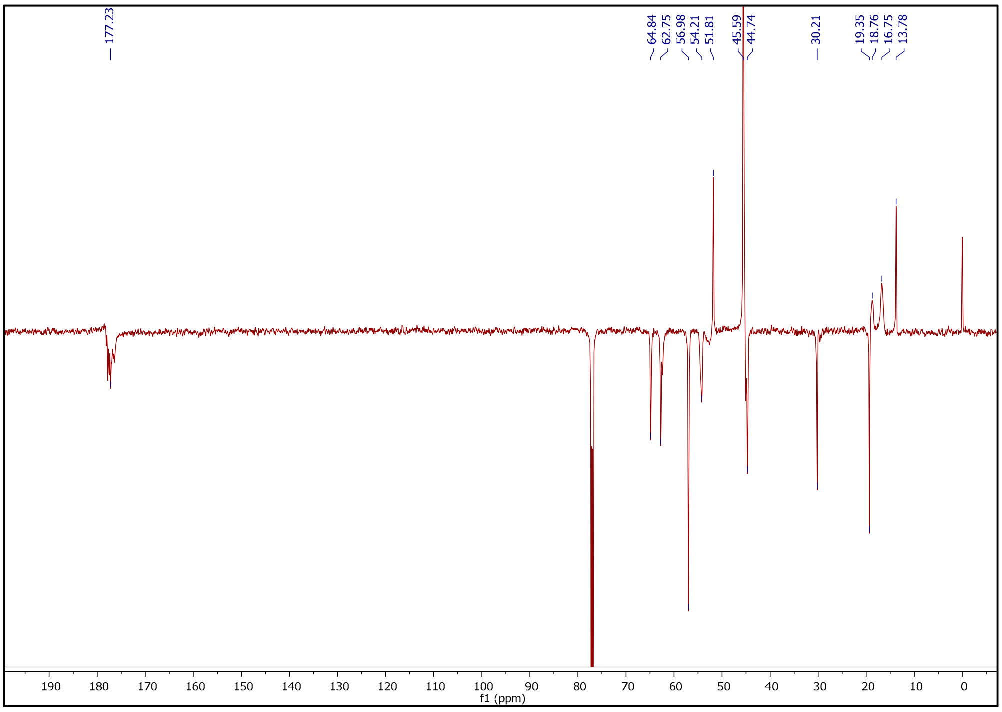

Figure S11. J-MOD NMR spectra (600 MHz, $CDCl_3$) of Eudragit® E 100.

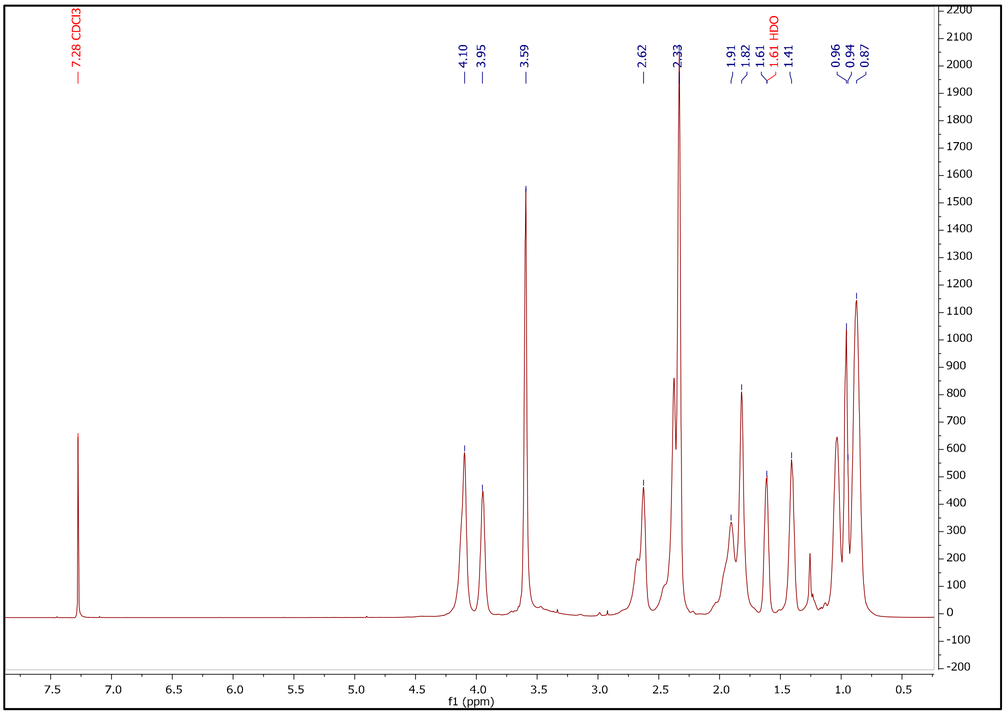

Figure S12. ^{1}H NMR spectra (600 MHz, $CDCl_3$) of Eudragit® E PO.

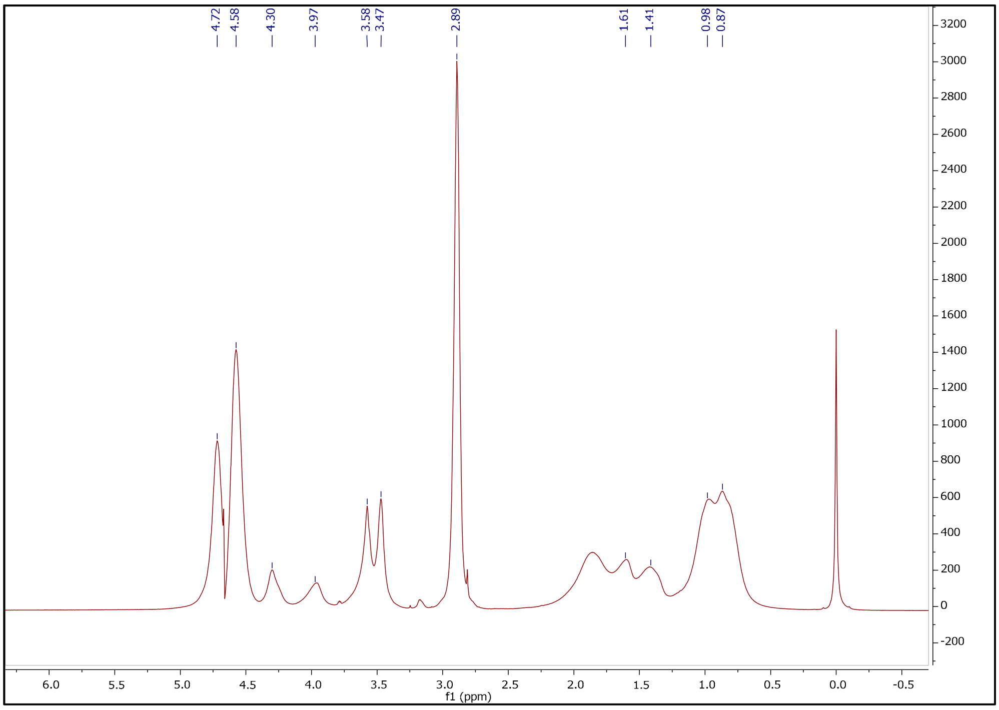

Figure S13. ^{1}H NMR spectra (600 MHz, D_2O) of EuCl-E-100.

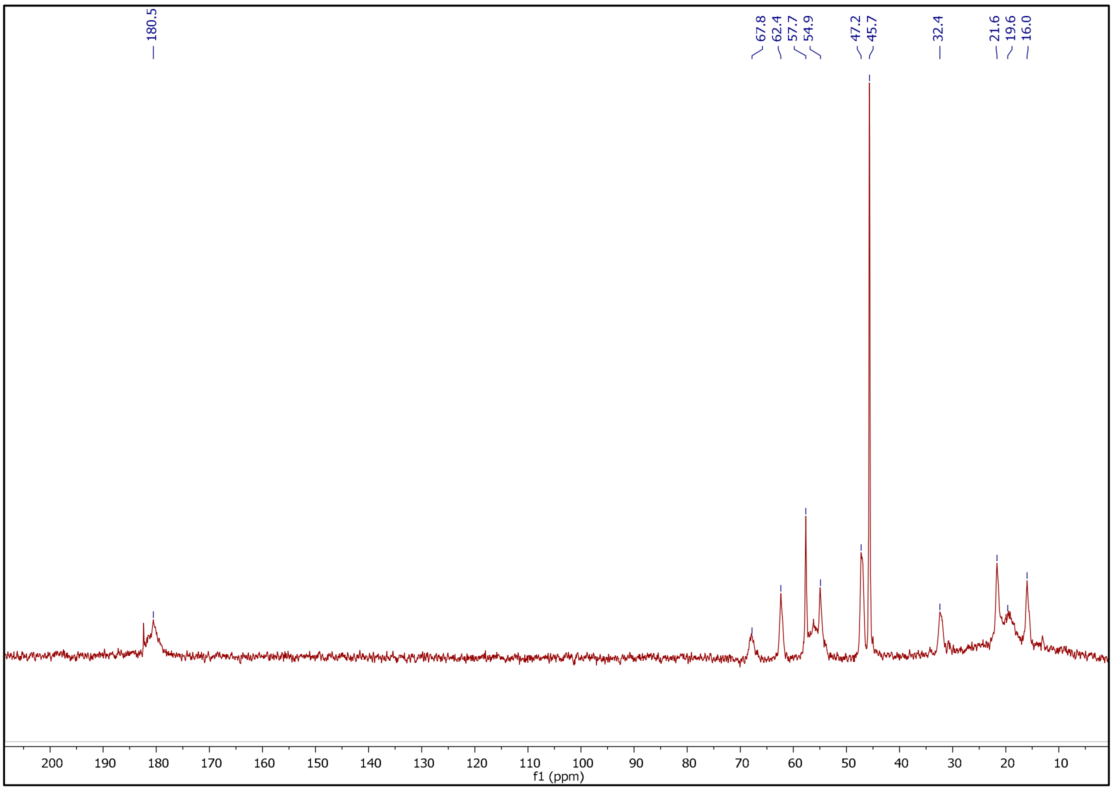

Figure S14. ^{13}C NMR spectra (600 MHz, D_2O) of EuCl-E-100.

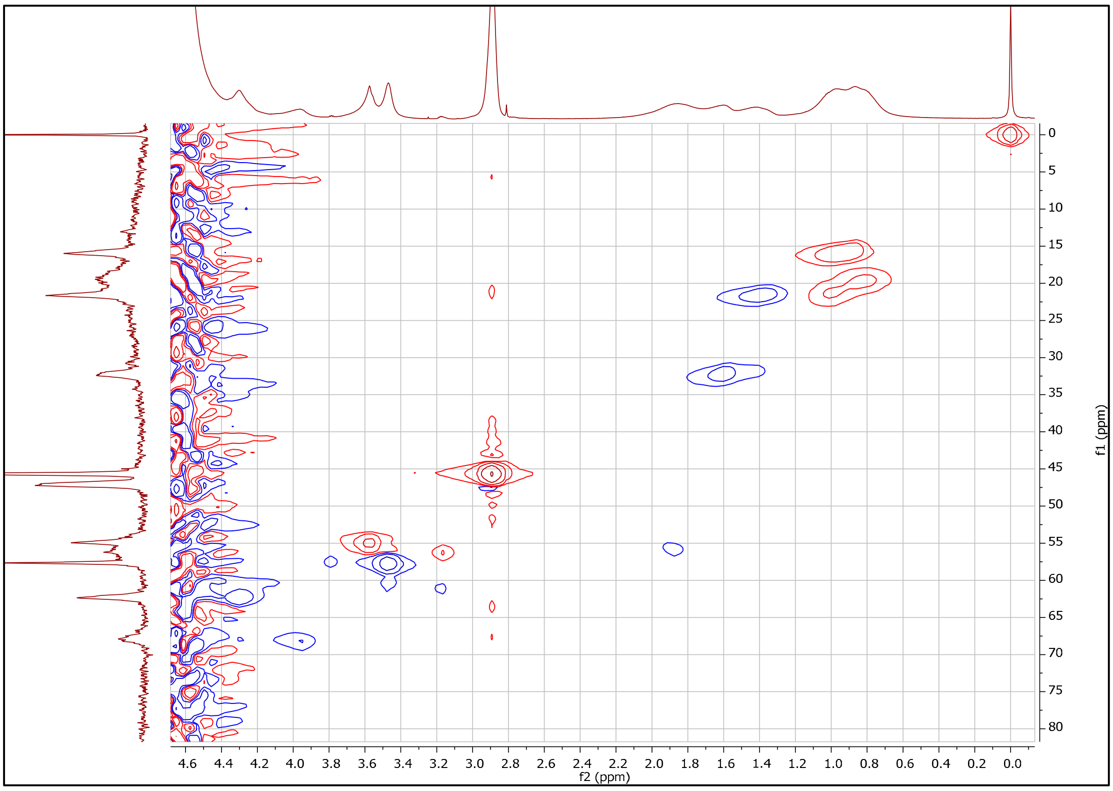

Figure S15. HSQC NMR spectra (600 MHz, $CDCl_3$) of EuCl-E-100.

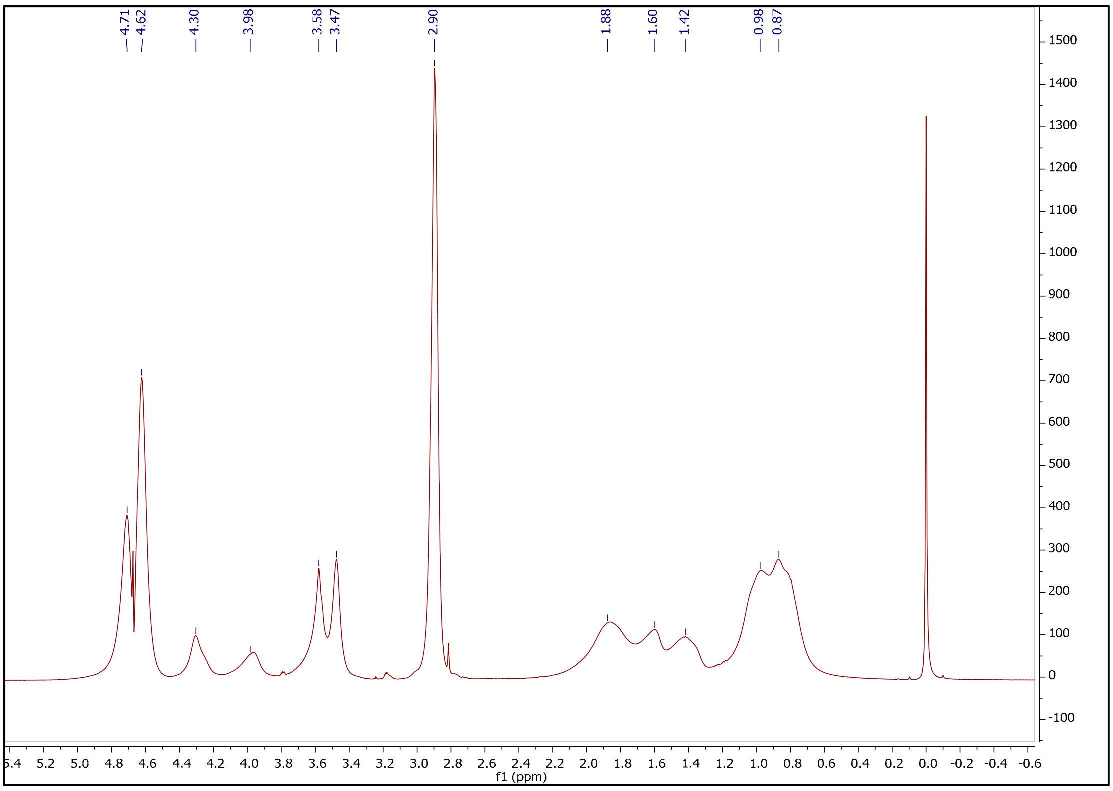

Figure S16. ^{1}H NMR spectra (600 MHz, D_2O) of EuCl-E-PO

4. Heating cycles (Cycle 3) in the DSC thermal analysis.

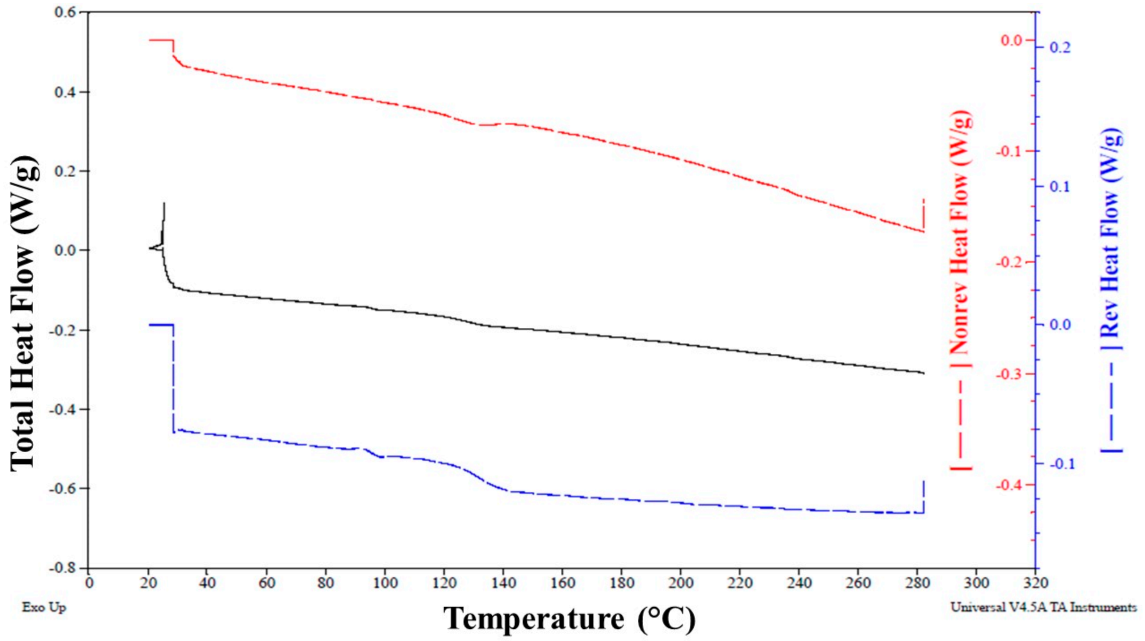

Figure S17. Cycle 3 (heating) for Eudragit® E100

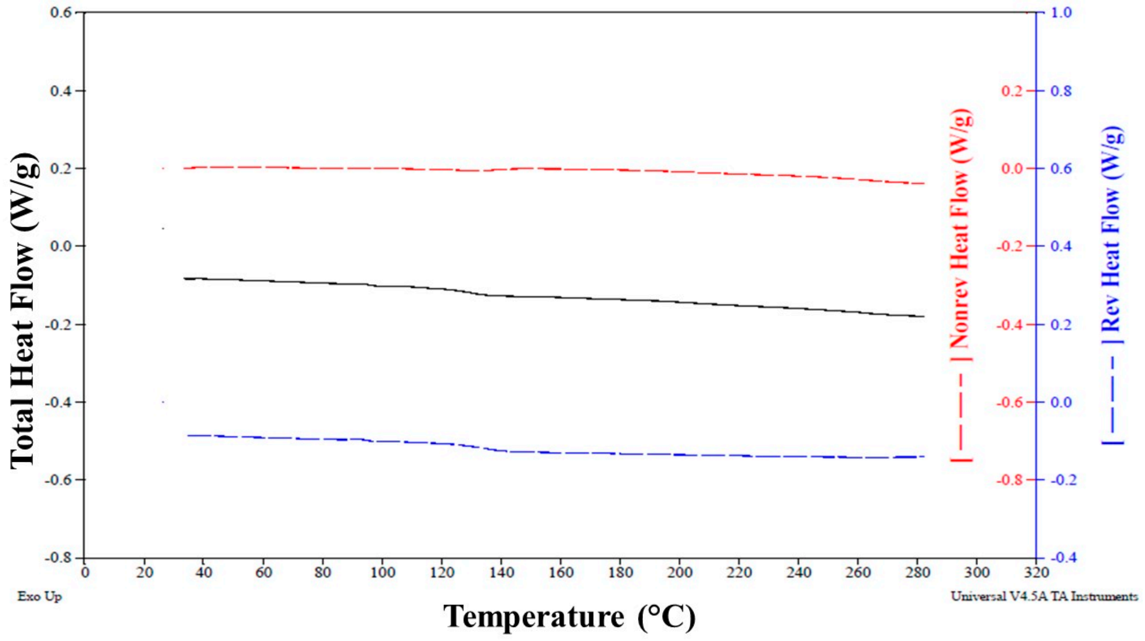

Figure S18. Cycle 3 (heating) for Eudragit® E PO

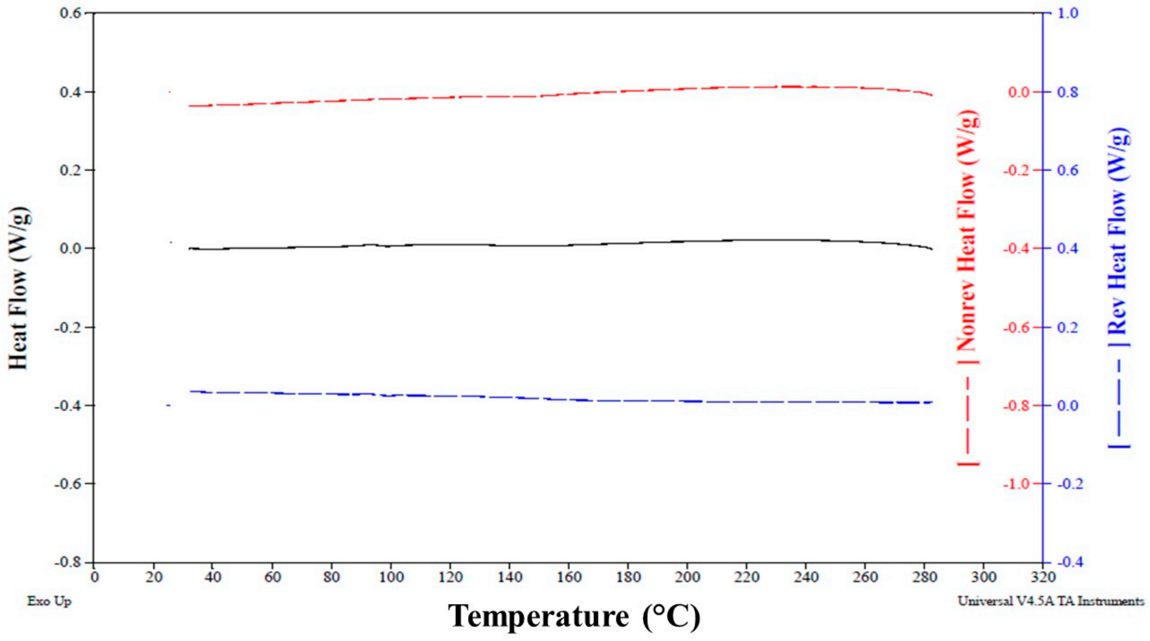

Figure S18. Cycle 3 (heating) for EuCl-E - 100

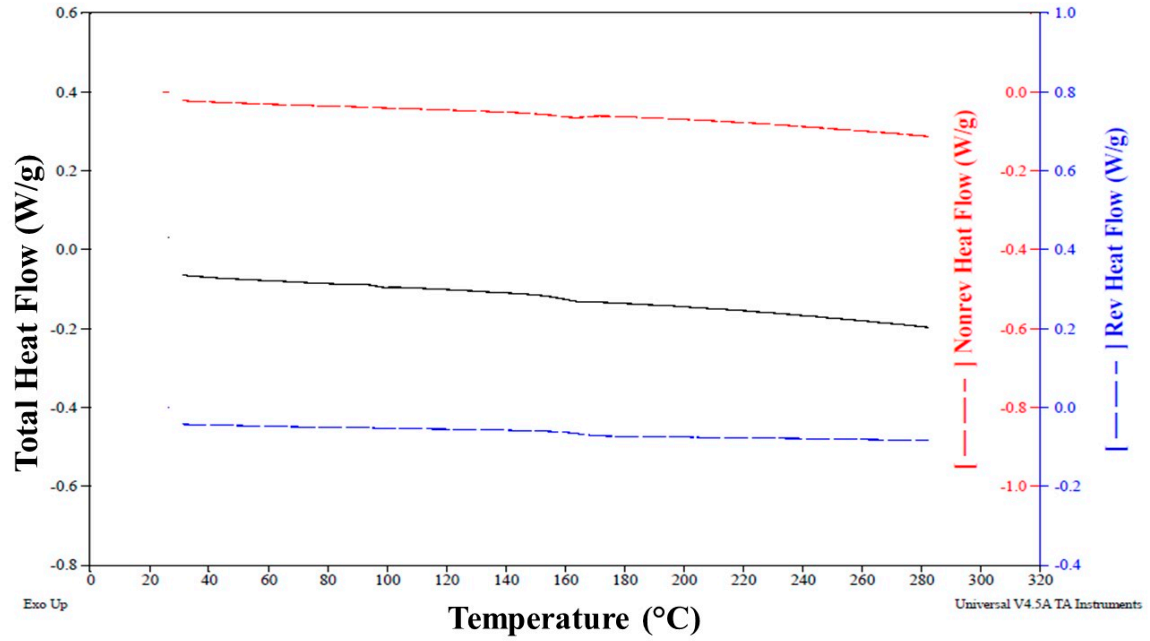

Figure S19. Cycle 3 (heating) for EuCl-E-PO

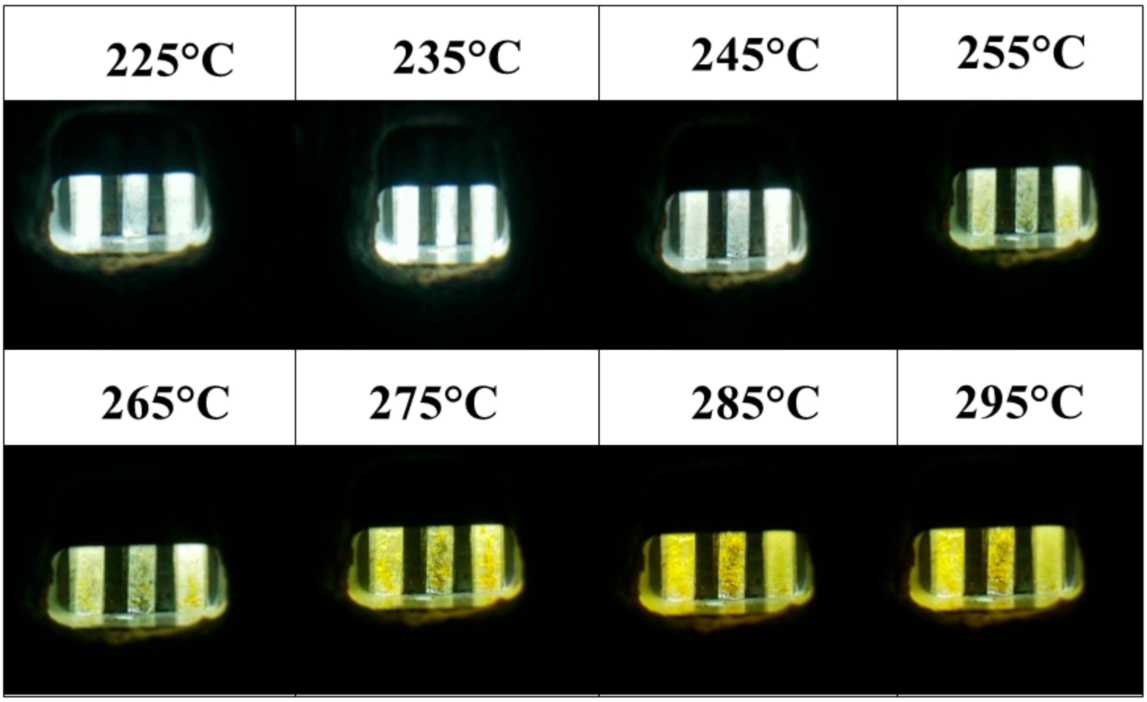

Figure S20. Representative scheme of the fusion / degradation process for the Eudragit E® polymeric materials and their processed forms. (Performed in capillary melting point apparatus).
